# Location, Location, Location: Signals in Muscle Specification

**DOI:** 10.3390/jdb6020011

**Published:** 2018-05-18

**Authors:** Chih-Ning Chang, Chrissa Kioussi

**Affiliations:** 1 Department of Pharmaceutical Sciences, College of Pharmacy, Oregon State University, Corvallis, OR 97331, USA; chanchih@oregonstate.edu; 2 Molecular Cell Biology Graduate Program, Oregon State University, Corvallis, OR 97331, USA

**Keywords:** myogenesis, mesoderm, WNT, FGF, BMP, SHH, RA, NOTCH, ephrins, sequence specific transcription factor

## Abstract

Muscles control body movement and locomotion, posture and body position and soft tissue support. Mesoderm derived cells gives rise to 700 unique muscles in humans as a result of well-orchestrated signaling and transcriptional networks in specific time and space. Although the anatomical structure of skeletal muscles is similar, their functions and locations are specialized. This is the result of specific signaling as the embryo grows and cells migrate to form different structures and organs. As cells progress to their next state, they suppress current sequence specific transcription factors (SSTF) and construct new networks to establish new myogenic features. In this review, we provide an overview of signaling pathways and gene regulatory networks during formation of the craniofacial, cardiac, vascular, trunk, and limb skeletal muscles.

## 1. Introduction

**Figure 1:**
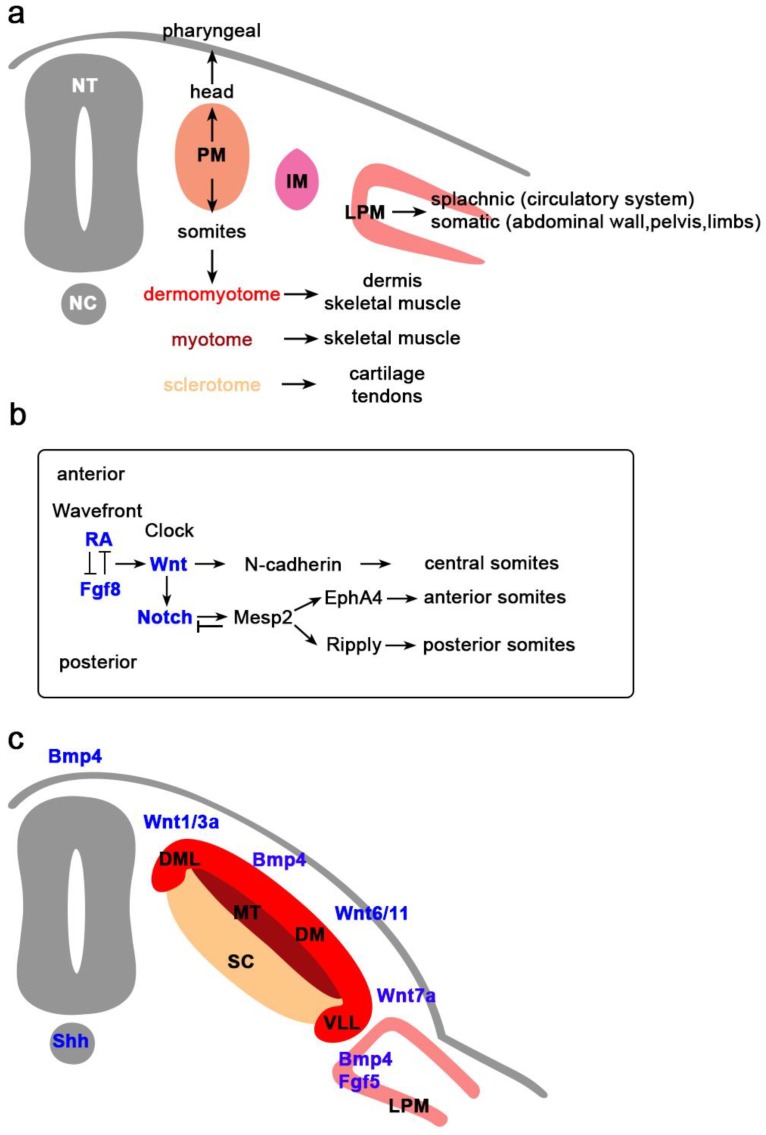
Signaling Molecules in Myogenesis (**a**) Paraxial mesoderm (PM) located next to the neural tube (NT). Dorsal PM gives rise to head and pharyngeal muscles while dorsal PM gives rise to somites. Intermediate mesoderm (IM) is located between the PM and the lateral plate mesoderm (LPM). LPM gives rise to the vascular system and skeletal muscles of the abdomen pelvis and some limb muscle. NC, notochord; (**b**) Anterior-posterior pattern of skeletal muscle is initiated by the RA/FGF8 inhibitory network, with AR promoting the anterior muscles and FGF8 the posterior. Activation of *Wnt* promotes the central somites and NOTCH signaling that will support formation of the anterior and posterior somites via EPH4 and RIPPLY, respectively; (**c**) Segmentation of somites into dermomyotome (DM), myotome (MT) and sclerotome (SC) is the result of signaling molecules secreted from the dorsal NT (BMP, WNT1/3a), notochord (SHH), ectoderm (WNT6/11, WNT7a, BMP4), and LPM (BMP4, FGF5). DML, dorsomedial lip; VLL, Ventrolateral lip.

In mammals, skeletal muscles begin to develop shortly after gastrulation and potentially can continue developing throughout their life. During gastrulation, epiblast cells ingress through the primitive streak to give rise to the three basic germ layers: ectoderm, mesoderm, and endoderm. During neurulation, as the primitive streak recedes posteriorly, ectodermal cells at the cranial end of the embryo proliferate, migrate, elevate, fold, and form the notochord and neural tube. Ectodermal cells shed and form the mesoderm, which further subdivides into cranial and trunk mesoderm. Early ingressing mesodermal cells migrate and populate underneath the ectoderm layer to form the lateral plate mesoderm (LPM), whereas the others subdivide into the paraxial (PM) (presomitic) and intermediate mesoderm. The skeletal muscle progenitor cells arise from the paraxial mesoderm, which flanks the axial mesoderm, notochord, and neural tube. Craniofacial muscles and cardiac muscle originate from the unsegmented PM, whereas, the skeletal muscles of body and limbs originate from the segmented PM that gives rise to somites (Figure 1a) [1].

The development of myogenic progenitors has been extensively investigated in vertebrate embryos since the 1990s using grafting [2], lineage tracing [3], in situ hybridization [4] and reporter (*LacZ*, *EGFP*) gene replacement [5,6,7] in both avian and mammalian embryos. These studies along with recent genomic wide association studies have established that the craniofacial, trunk, and skeletal musculature have unique anatomical origins and molecular networks [8,9,10,11]. Signals like retinoic acid (RA), sonic hedgehog homolog (SHH), bone morphogenetic proteins (BMP), WNT, fibroblast growth factor (FGF) are emitted from the neural tube, notochord, ectoderm, dorsal laminae, and neural crest [11]. RA and FGF set up the anterior-posterior cues respectively in early gastrulation. High concentration of RA triggers the formation of cranial PM and initiates the expression of cardiac (*Pitx2*, *Tbx1*, *Pax7*) and cranial (*Nkx2.5*, *Tbx5*, *Gata4*) SSTF networks [12,13]. FGFs from the tail end of embryo antagonize RA activity and initiate the formation of trunk PM, segmentation, and somite formation. BMP, SHH, WNT, and NOGGIN promote dorsal-ventral and medial-lateral cues that mediate trunk and abdominal SSTF networks (*Six1/4*, *Pax3*, *Pax7*, *Pitx2*) that in turn regulate the expression of basic helix-loop-helix (bHLH) myogenic regulatory factors (MRF; *Myf5*, *Myf6*, *Myod1*, *Myog*) [14]. MRFs regulate the commitment, determination, and differentiation of muscle progenitor cells [15]. MRFs act redundantly but control the cell-autonomous activation of myogenesis and regulate the expression of genes involved in contraction [16,17,18,19,20]. MYF5 and MYOD define the committed myoblasts, whereas MYF6 and MYOG define the differentiated myocytes. Differentiated myocytes exit the cell cycle and undergo morphological changes. In this review, we discuss the signaling pathways and gene networks that control skeletal muscle development in head and trunk, in addition to vascular and cardiac muscle (Table 1).

## 2. Craniofacial Myogenesis

Sixty muscle groups provide support for seeing, feeding, breathing, and moving of the head and neck. These muscles originate from the cranial mesoderm as a part of the paraxial mesoderm. Cranial mesoderm later specifies into the prechordal, pharyngeal, and lateral mesoderm. Prechordal mesoderm gives rise to the extraocular muscles (EOM) which encompass six muscles that control the eye movement [87]. Pharyngeal mesoderm forms the branchial arches (BA) that give rise to the masticatory, facial, pharyngeal and laryngeal muscles, and the secondary heart field [88,89]. Part of the pharyngeal mesoderm contributes to splanchnic mesoderm, that populates the tongue, axial neck, and heart (Figure 1a).

**Figure 2:**
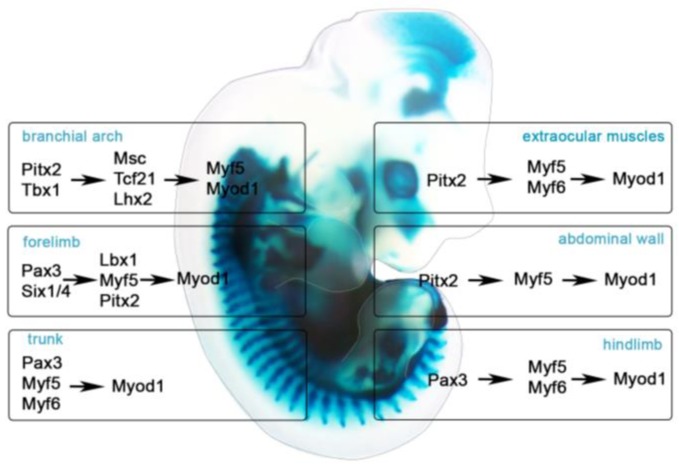
Gene Networks Involved in Muscle Specification. LacZ staining of E12.5 *Pitx2^LacZ/+^* mouse. Extraocular muscles (EOM) and abdominal wall muscles are specified by PITX2. Branchial arch muscles are specified by PITX2 and TBX1. Forelimb, trunk and hindlimb muscles are specified by PAX3.

Craniofacial myogenesis is influenced by RA, BMP, FGF, and WNT and BMP inhibitors. RA activates *Pitx2* and inhibits *Tbx1* expression in the anterior head mesoderm. As RA is weakened in posterior head mesoderm, RA antagonist, FGF8, activates *Tbx1* expression [90,91,92]. FGF and BMP reinforce TBX1 activity and activate *Alx4* and *Msc* expression. FGF signaling spreads posteriorly and establishes the mature head mesoderm patterns, drives *MyoR* and *Tbx1* expression in pharynx, BA muscles, and heart [93]. Grafting experiments in chick embryos have indicated that WNT1 and WNT3A from the dorsal neural tube, WNT13 from the surface ectoderm, and BMP4 and BMP7 from the dorsal neural tube and ectoderm inhibit head muscle myogenesis [94,95]. However, the WNT antagonists, FRZB, and the BMP inhibitors, NOGGIN and GREMLIN, from the cranial neural crest and other surrounding tissues, induce craniofacial myogenesis [95]. Lineage tracing approaches in both avian and mouse models demonstrated that EOM and BA cells contribute to distinct muscles [96]. Homeodomain transcription factors *Tbx1*, *Pitx2*, *Tcf21* and *Lhx2* are expressed in the pharyngeal mesoderm and regulate cranial myogenesis. PITX2 specifies EOM by activating *Myf5*, *Myf6*, and *Myod1* [14]. During BA myogenesis, PITX2 activates *Tbx1*, *Msc*, *Tcf21*, and *Six2* [97,98]. TBX1 induces the expression of *Fgf* receptors and their ligands. Perturbation of *Fgf* in mice or fish leads to hypoplastic and asymmetric muscles [99,100]. TBX1 and FGF activate *en2* in myogenic cells in the dorsal mandibular pharyngeal arch and contribute to jaw development. Although function of EN2 is still unclear, studies in chick embryos implied that signaling pathways prevent prematurely specification. TBX1, MSC, and TCF21 together activate MYF5 and lead to MYOD1 and MYOG expression (Figure 2) [101]. PAX7 is expressed in MRF^+^ cells [88,94,102] and controls the formation of head satellite cells [103]. Head satellite cells derive from the MESP1*^+^* cells [96] but only the EOM and pharyngeal satellite cells express ISL1, ALX4 PITX1, PITX2, TCF21, cytokines, and chemokines [14,96,104].

Tongue [105] and neck muscles arise from the medial-dorsal and lateral-ventral domains of the occipital and cervical somites [106]. WNT signaling regulates tongue myogenesis [107] and differentially activation of MYF5 and MYOD1 [108]. WNT from the neural tube induces the expression of *Colloid-like1*, and reinforces BMP activity in the most anterior medial dermomyotome (DM) and regulates neck myogenesis [31].

## 3. Cardiac Myogenesis

Cranial mesoderm cells ingress laterally and contribute to the formation of the myocardium and endocardium [109], while medial and lateral splanchnic mesoderm give rise to the chambers of the heart [97]. RA establishes the posterior boundary of the secondary heart field [110], influences the development of the posterior and sinoatrial region [111] and transforms the cranial cells into cardiomyocytes [112]. BMP is expressed at the posterior and lateral region of the embryo and recruits head mesoderm cells to the heart [109,112]. WNT1, WNT3a, and WNT8c from the posterior primitive streak and lateral mesoderm inhibit cardiogenesis [113] and define the migratory limits of the cardiomyocytes. BMP antagonist NOGGIN from the notochord provides timely control to initiate early heart formation [12,13]. Collectively, the above signals promote expression of NKX2.5, TBX5, MEF2C and GATA4 [12,13] and the chromatin remodeling subunit SMARCD3 [114] and force the transition of mesodermal cells into cardiocytes [115].

Just after gastrulation, the cardiac crescent or 1st heart field, forms a tubular structure, with an outer myocardium and an inner endocardium. The linear heart tube connects with the artery at the anterior pole which forms the outflow tract and with a vein at the venous pole, which forms the inflow tract [116]. BMP2 activates NKX2.5 expression in the cardiac crescent. NKX2.5 interacts with GATA4 and TBX5 [117] to promote cell differentiation [118] and suppress the expression of FGF10 to prevent prenatal development [119]. BA-derived cells, 2nd heart field, express FGF8, FGF10, ISL1 [120] and TBX1 [121] proliferate, contribute to the extension of the tubular heart, and the formation of outflow tract and right ventricle. PITX2 activates MSC [97,98] and TBX*1* [122], which triggers FGF10 expression to stimulate cardiac muscle progenitors towards migration and proliferation [123]. Cranial neural crest (NC) cells contribute to the formation of valve cushions of the outflow tract [124,125]. Histone deacetylases (HDACS) [126] and TGFβ modulate their migration [127] throughout the dorso-ventral axis [128,129]. TGFβ represses cardiomyocyte specification to prevent premature differentiation [130].

## 4. Smooth Muscle Formation

Smooth muscle cells (SMC) give rise to gastrointestinal, urogenital and respiratory tract, and blood vessels [131]. The specific mechanisms responsible for SMC determination and differentiation are largely unknown. A heterogeneous population of cells derive from somites [132], secondary heart field [133], splanchnic mesoderm [134], and NC cells [135] contribute to the vascular SMC formation. BMP, NOTCH, SHH, and TGFβ1 promote vascular SMC specification and differentiation by activating serum response factors (SRF) and their cofactors. Activation of SRF leads to expression of ACTA), SM myosin heavy chain, and SM22 (review [136,137]). BMP and NOTCH initiate myogenic patterning in vascular SMC development. BMP2 stimulates their migration and the expression of the Va myosin [138], whereas NOTCH is required for differentiation of the vascular SMC progenitors [139]. SHH regulates vascular SMC proliferation and the formation of coronary vessels [140]. TGFβ1 inhibits vascular SMC growth, increases proliferation of the NC-derived vascular SMC via plasminogen activator inhibitor (PAI) [141]. TGFβ1 also stimulates vascular SMC differentiation by activating ACTA2 [142], SM22 [143], SMAD3 [144], and RhoA [145]. TGFβ/BMP co-receptor ENDOGLIN, a descendent of the PAX3^+^ vascular SMCs is required for angiogenesis [146]. More recent studies also indicated the PAX3^+^ DM might act as a stem cell niche for vascular SMCs [147].

## 5. Trunk Myogenesis

Skeletal muscles of the trunk originate from somites, which are derived from the segmented PM and located on the either side of the neural tube. Somitogenesis occurs progressively in an anterior to posterior sequence, simultaneous with regression of the primitive streak soon after neurulation (Figure 1a). After somites are formed and segmented, cells start to differentiate along the dorsal-ventral axis [1]. Ventral cells undergo an epithelial to mesenchymal transition to form the sclerotome, which will generate cartilage and associated connective tissues of the vertebrate and ribs. Dorsal cells remain epithelial and form the DM, which will generate the dermis, skeletal muscle of the truck and limbs, and brown fat [148]. The first myogenic tissue, myotome, arises later from the dorsomedial (DML) and ventrolateral (VLL) lips of the DM [149]. The DM is subdivided into the lateral hypaxial and medial epaxial DM. Cells of the VLL form the hypaxial myotome that gives rise to the lateral trunk and limb musculature, and cells from the epaxial myotome give rise to the deep muscles of the back (Figure 1c) [150,151].

### 5.1. Somitogenesis

Cells from the anterior tip of presomitic mesoderm undergo different morphogenetic changes and form as an epithelial ball, termed somitomeres [152,153]. Somitic cells are temporally controlled by the cyclin expression of intrinsic oscillating genes (“clock”) that set the pace [154] and signals secreted from nearby tissues (“wave front”) that define the position of the posterior border of each new somite [155,156]. Clock genes are expressed cyclically to define the time interval. Combinatorial signals (RA, FGF8, WNT3, and SHH) regulate the region where the clock genes are segregated and gives rise to a new pair of somites. Gradient signals provide the spatial clue and define the boundaries of segmentation of newly formed somites. FGF8 and WNT3 are produced in the caudal region of the embryo, RA is secreted by the cranial region of newly formed somites [152,153,157], and SHH is released from the notochord [155]. SHH signaling is required to preserve the FGF8 gradient. FGF8 signaling counteracts an opposing RA gradient [158]. The interaction of WNT and FGF8 regulates not only the separation, but also the structure of somites. At the anterior end of PM, WNT induces the expression of *ß-catenin* and *N-cadherin* in the center of somite. N-cadherin regulates the adhesion of the epithelial surface of the somite by forming tight junctions and a basal lamina that separates it from nearby tissues [64]. At the same time, WNT induces its antagonist, NOTCH, at the posterior end of somite, while NOTCH induces *Epha4* and either *ephrinB2* (chick) or *Ripply* (mouse). Together, they define the posterior and anterior end of the somite and promote their separation (Figure 1b) [64,159,160].

Mature somites differentiate into different compartments and determine the fates of cells (Figure 1c). Compartmentalization is induced by SHH and NOGGIN from the floor plate of neural tube and notochord, and by WNTs and BMPs from the ectoderm and dorsal laminae of the neural tube. Noggin binds and inactivates Bmp4 and creates morphogenic gradients [161,162]. Cells at the ventromedial part of the somite, located closer to the notochord and the ventral part of neural tube, receive a higher concentration of NOGGIN and SHH. As they proliferate, they lose the expression of N-cadherin and later become mesenchymal cells and develop into sclerotome. They express PAX1 and SOX9 that constitute the cartilage, vertebrae, and ribs and I-MF that inhibits muscle differentiation. Cells located at the dorsomedial part of the somite receive a low concentration of NOGGIN and SHH, remain epithelial, and give rise to DM [163,164,165]. The epithelial DM is divided into dorsomedial (epaxial), central (dermatome), and ventromedial (hypaxial) DM. The epithelial DM is influenced by different signals to activate distinct regulatory gene networks that contribute to specific muscle formation in the body. PAX3 marks the myogenic lineage in the DM. The PAX3^+^ cells mark the DM-derived cells that give rise to skeletal [166,167] and vascular muscles [168,169]. Ablation of *Pax3* leads to the absence of forelimb muscles [170,171]. SIX1, SIX4, and their cofactors EYA1 and EYA2 directly bind on the promoter of *Pax3* and activate its expression [15,172,173]. Double mutants of *Six1/4* and *Eya1/2* exhibit loss of PAX3 expression that leads to migratory defects in hypaxial muscles [172,173].

PAX3 plays a key role in activating the MRF expression. MYF5 is expressed earlier in the dorsomedial lip of DM and then in the hypaxial myotome under the influenced of WNT1 and SHH to give rise to the epaxial muscles [174,175]. MYOD1 expression is induced by WNT7a and inhibited by BMP4 in the ventrolateral DM. Expression of MYF6 and MYOG coincides with the innervation and myofiber formation [18,19].

### 5.2. Epaxial Muscle Formation

Cells in the epaxial DM receive WNT1 and WNT3a from the dorsal neural tube and low levels of SHH from the floor plate of the neural tube. WNT1 and WNT3 activate *Myf5* [176] and *Myod1* expression through the WNT canonical pathway [177,178,179,180,181]. Low level of SHH contributes to *Myf5* expression through the GLI2 and ZIC1 [182,183]. Epaxial DM receives NOTCH/DELTA1 signaling from migrating NC cells and induces MYF5 expression [184]. PAX3 also regulates *Myf5* expression through direct binding to *Dmrt2* motifs [15,181,185]. PAX3 and MYF5 trigger MYOd1 expression, which drives myoblast differentiation [175]. MYF6 is expressed in the somites simultaneously with MYF5, although what activates its expression is still unknown [20].

### 5.3. Dermatome Formation

The dermatome is heterogenic and distinct, marks the boundary between the epaxial and hypaxial DM, and gives rise to the dermis of head and neck, satellite cells, and brown adipocytes [9,151,186,187,188]. The combined influence of SHH from the notochord, WNT1 from the dorsal neural tube, WNT11 from surface ectoderm and dermatome, and the negative influenced of BMP4 from the LPM marks the epaxial-hypaxial border. Homeobox gene *engrailed1* (*en1*) blocks the epaxial-hypaxial interface. EN1 is expressed on the border of the hypaxial maker SIM1, which marks the territories of motor neurons and regulates muscle patterning [189]. The EN1-SIM1 expression boundary marks the epaxial-hypaxial DM boundary and the dermatome. The specification of cells in the dermatome is diverse. A subset of cells in the medial part of dermatome receives BMP from the roof plate of the neural tube and migrates dorsally into the sub-ectodermal space to form cartilage and the medial margin of scapula in birds and mammals [190,191,192,193]. Another subset of cells, which is under the influence of *Wnt6* from the dorsal ectoderm, expresses PAX3 and PAX7 and forms the satellite cells [9,151,186,187,188]. PAX3 induces the expression of PAX7 in the central DM. The PAX3^+^/PAX7^+^ muscle progenitor cells populate the myotome [167,187,188] and receive *neurogulin1* (*nrg1*) from the migrating NC cells through NRG1 receptor ERBB3. The NRG1-ERBB3 signaling maintains PAX7 expression and prevents premature myogenic differentiation [194]. In addition, DERMO-1 also modulates the development of mesenchymal cell lineages including muscle and dermis [195]. DERMO-1 represses transcriptional activity of MEF2 and MYOD in a dose-dependent fashion [196].

### 5.4. Hypaxial Muscle Formation

The hypaxial DM gives rise to vertebral muscle, the diaphragm, abdominal muscles and muscles of the limb. Development of hypaxial DM is highly influenced by the LPM [165,197]. Upon receiving WNT7a from the dorsal ectoderm and Bmp4 from the LPM, cells give rise to hypaxial myotome [165,197]. BMP signaling induces somite-derived endothelial cell differentiation and migration via VEGFR2 expression [198,199], whereas the WNT7a/ PKC activates Pax3 in hypaxial DM [200]. PAX3 is critical for the onset of embryonic myogenesis by regulating delamination via C-MET, migration via LBX, and determination of muscle progenitors [166,167] in the limb [201,202,203,204,205]. PITX2 lies genetically downstream of PAX3 in the hypaxial DM [206,207]. Activation of *Pitx2* regulates expression of MYOD1 and MYOG, myoblast motility, and skeletal muscle maintenance [208,209]. PAX3 regulates the expression of ITM2a in DM, limb buds, adult muscle fibers, and satellite cells [210]. In turn, ITM2a regulates muscle creatine kinase (CK-M) expression and muscle differentiation [211].

### 5.5. Myotome Formation

Myotome is the first myogenic structure located between the DM dorsally, and the sclerotome ventrally. Cells of the DM, lose expression of N-cadherin, delaminate, and translocate underneath the DM to form the new structure, the myotome. Surrounding signals trigger the expression of *MRFs* and establish their myogenic identity as they populate the myotome. Upon arrival, the cells start to differentiate and fuse to become myofibers.

During the first wave of myogenesis, myogenic progenitors from the DM develop into mononucleated myocytes. Cells from the dorsal part of myotome originate from the dorsomedial lip (DML), migrate ventrally as they receive WNT1 and WNT3a from the dorsal neural tube, WNT7a from dorsal ectoderm, and SHH from the ventral region of the neural tube and notochord. The WNT and SHH signaling defines the myogenic properties of migrating muscle precursors by activating the expression of MYF5 [72,180,182] and MYOD1 [148]. Although the cells from the central DM contribute to the myotome, they are less significant than cells from the DML and ventrolateral lip (VLL). Cells in the central DM receive neurophrin-3 and WNT1 from the neural tube and become part of the myotome [212]. Cells from the VLL are essential for lateral extension of the myotome [213]. They receive WNT7a from the dorsal ectoderm and BMP4 and FGF5 from the LPM, inducing the expression of MYOD1, but not MYF5 [214].

After entering the myotome, MYF5 and SHH together activate the expression of *Fgf4*, which regulates the proliferation and differentiation of the *MRF* expressing myocytes [215]. Once the myocyte identity is established, they express PAX7 before undergoing terminal differentiation. SHH is crucial to maintain the expression of PAX7 in migrating MYF5^+^/MYOD1^+^ cells. Interfered by SHH results in accumulation of PAX7^+^ cells and a small myotome in chick and mouse embryos [216,217]. Myocytes receive WNT11 as they elongate along the somites and express both slow- and fast-type cytoskeletal proteins, including MYH (slow type I), MYH3 (embryonic), ACTC1, ACTA1, DESMIN, JAG2, ß-ENOLASE, and CA2 [8,217,218]. Myocytes fuse and form the first multinucleated MyHC^+^ myofibers spreading from the ventral to the dorsal myotome [217]. *Six1* and *Six4* expression activates the fast muscle program [218].

### 5.6. Limb Muscle Formation

Limb muscle development is a triphasic process. During embryonic myogenesis in mice (E9-E12), the first multinucleated muscle fibers are formed from PAX3^+^ embryonic myoblasts, followed by PAX3^+^LBX1^+^C-MET^+^ migratory myoblasts that provide the basic pattern and primary myofibers throughout the limbs. During fetal myogenesis (E13-E16), the PAX3^+^/PAX7^+^ fetal muscle progenitors use embryonic fibers as a scaffold to expand the muscle mass. During perinatal myogenesis (E17-P5), PAX7^+^ muscle progenitors (satellite cells) are located between the basal lamina and the fiber plasma membrane and remain mononucleated and quiescent. After muscle damage, satellite cells can activate, fuse with existing myofibers, and repair damaged muscle (Figure 2) [10,219].

Embryonic (primary) myogenesis begins when the first embryonic myoblast progenitors of the hypaxial DM start to delaminate and migrate to forelimb buds [220]. PAX3 activates expression of c-MET and LBX1, and the PAX3^+^LBX1^+^c-MET^+^ cells delaminate, become mobile, and start to migrate distally [203,204,205]. LBX1 is crucial for the migrating embryonic myoblast progenitors (EMP) [205,221]. The migratory directions are controlled by signals from the surface of limb buds, zone of polarizing activity (ZPA). Misrouted EMPs either migrate slowly or accumulate in the mesoderm of ventral body wall and form smaller or no muscles [222,223]. SHH signals from ZPA regulate the migratory patterns of EMPs, maintenance of limb bug outgrowth, dorso-ventral limb patterning, and development of skeleton, cartilage, and tendons of digits [224,225]. SHH and FGF*s* from the ectodermal ridge control the expression of HGF/SF, the only known ligand of c-MET [226], which directs the migration of EMPs [203,204]. Migratory EMPs also express the chemokine receptor CXCR4. This receptor responds to chemo-attractant SDF1 from the limb buds, which also directs the migratory routes [227]. SF/HGF, BMP, WNT and SFRP-2 inhibit prematurely differentiating EMPs and allow them to multiply and populate the limb bud parallel to establishing the myogenic program [198,228]. The Wnt/ß-catenin/PITX2 pathway controls motility and proliferation of the EMPs by regulating expression of the growth control genes *Ccnd1*, *Ccnd2* and *c-myc*, [209]. IGF contributes to the proliferation and determination of EMPs [229]. IGF-1 and IGF-2 play a role in PAX3 and MYOG expression via PI3K and MYOD through FGF18 [230]. Upon their entry into the limb bud, EMPs express high levels of MRFs and become embryonic myoblasts and myofibers. At the same time, SHH initiates the expression of MYF5 though GLIA [231]. The spindle-shaped EMPs receive FGF and TGFβ, and increase in cell number while they align to each other. FGFs maintain the expression of MSX1 to prevent their premature differentiation. The newly formed MYOD1^+^embryonic myoblasts exit the cell cycle and express MYOG and MEF2 [232]. During differentiation, embryonic myoblasts undergo several changes, including cell aggregation, elongation, metabolic changes, cell membrane fragmentation and cytoskeleton assembly. They start to express MyHC and myosin light chain 1 (MYL1), secrete fibronectin, RGD-binding integrins, cadherins, and cell adhesion molecules that promote the alignment of migratory cells [233,234,235], and fuse to each other to become multinucleated primary myofibers [236].

Fetal myogenesis begins when a subset of PAX3^+^ myogenic progenitors start to express PAX7 while their PAX3 expression is decreased in the central DM [167,188,237,238]. Mouse *Pax7* mutants exhibit defects in fetal myogenesis with smaller muscles, fewer myofibers, and impaired satellite cells, leading to defective muscle regeneration [239,240,241,242,243]. PAX7^+^ cells constitute the fetal myogenic progenitors (FMP). FMP receive TGFβ, BMP, and WNT/β-catenin signaling from the ectoderm and the dorsal laminae to block the premature differentiation. TGFβ*2* from the ventral region of the limb buds and later from the muscle mass [173] represses MYOD1 and MYOG activity [244], induces FMP migration and inhibits their differentiation [245,246]. They increase their number and contribute to all muscles [237] as they migrate and fuse to the primary fibers and each other to form the secondary fibers. PAX7 directly binds and activates *Nfix*, which marks the fetal myoblasts. NFIX represses the embryonic muscle genes *Sox6* and *MyHCI*, and activates the fetal muscle genes *α7-integrin*, *β-enolase*, muscle creatine kinase, and muscle sarcomeric proteins [247]. PITX2 and PITX3 are also expressed in fetal myoblasts [248] as they commit to the fetal program and become myocytes by subsequent expression of MYOD1, MYF5, and MYOG [167]. When differentiated, fetal myocytes fuse to embryonic myofibers and give rise to doughnut-shaped, multinucleated fetal myofibers [188,249].

During birth and the first few weeks after birth, the third wave of myogenesis begins with an increase in number and size of myofibers. PAX7^+^satellite cells express myogenic (c-MET, HGF/SF, MSX1) and endothelial (CD34) markers [250,251]. Expression of *c-met* and *Hgf/Sf* promotes delamination and migration from the basal lamina once activated in response to stress. Activated satellite cells turn off *Pax7* and begin to express *Myod1* and cell-cycle markers and undergo multiple rounds of asymmetric cell division. A small number of activated satellite cells returns to an undifferentiated state (PAX7^+^MYOD1^-^) for self-renewal. PITX2 regulates satellite cell division by stimulating the expression of *Ccnd1*, *Ccnd2* and *Myf5*, and downregulates *miR-15b*, *miR-106b*, *miR-23b*, and *miR-503* [252]. Daughter satellite cells express MYF5 and MYOD, and fuse to pre-existing myofibers to form new fibers [238]. MSTN, a member of TGF family, regulates satellite cell renewal and muscle growth [253]. MSTN inhibits *Pax7* expression and binds to activin. Activin activates ALK4/5, mediates SMA2/3 phosphorylation [254,255], and recruits SMAD4. MSTN/activin/SMAD signaling pathway inhibits MRF expression [256], and limits proliferation and differentiation into myofibers via AKT/TORc1/P70S6K pathway [257,258,259]. MSTN antagonist, Follistatin (*Fst*), promotes PAX*7* activity [260,261,262], regulates MRFs expression and muscle fiber formation [263]. Bone morphogenetic protein and activin membrane-bound inhibitor (BAMBI), another member of TGF family, responds to Wnt/β-catenin pathway and plays a role in regulating SMAD activity [264].

## 6. Abdominal Myogenesis

Abdominal muscles encompass three layers of skeletal muscles, which merge toward the midline and form a sheath to assist breathing and protect the inner organs. Abdominal muscles derive from LPM and somites. LPM gives rise to somatopleure (lateral) and splanchnopleure (medial). The somatopleure and the surface epithelial endoderm give rise to the body wall in three waves similar to trunk myogenesis (Figure 1a) [265,266]. Myogenic cells within a thin sheet of LPM-derived cells proliferate without delamination, remain within the DM epithelium, and migrate ventrally into the somatopleure to cover the abdomen and form the primary body wall [266,267]. The primary body wall is composed of myogenic and epithelial cells that provide the first tissue to cover the endoderm. After several days, a second and third wave of myogenic progenitor cells move into the somatopleure and all layers are joined at the midline [268,269]. The secondary body wall forms muscle, skin, ribs, and sternum. Cells of the secondary body wall are heterogeneous, composed of myogenic, mesenchymal and NC cells. WNT/PITX2 signaling plays a role in the abdominal myogenesis. WNT/β-catenin signaling instructs mesodermal specification of somites and LPM-derived mesenchymal cells in ventral body wall as a paracrine signal [270]. WNT7a from the dorsal ectoderm and BMP from the LPM activate expression of PAX3 and its targets. WNT signaling activates *Pitx2* expression in LPM. PAX3^+^PITX2^+^ cells proliferate, migrate to the midline, and express *MRFs* [271]. Ablation of *Pitx2* results in repression of *T-box* and activation of *Hox9-11* genes in the abdominal wall [266,272]. AP-2a regulates the closure of all four body folds by regulating the epithelial-mesenchymal interactions and cell-cell communication from the ectoderm to the abdominal mesoderm [268,273]. Defects in these pathways lead to hypoplasia in the abdominal wall or organ exposure. HOXb2 and HOXb4 activate ALX3/4 [274,275] and regulate the primary ventral body formation [276,277,278]. Independent from the PITX2 pathway [279], BMP1 might enhance the activity or availability of TFGß5^+^ myofibroblasts during the first wave [280] and initiate the migration in the second wave [281]. TGFβ2/3 plays a fundamental role in activating cytoskeletal components (*Tagln*, *α-Sma* and *desmin*) that support myofibroblast migration towards the midline [282]. The signaling pathways contribute to ventral wall closing by involving the assembly and function of extracellular matrix and collagen fibrils [283]. NC cells migrate to the midline and contribute to the second body wall formation. AP-2 is expressed in migrating NC cells [284].

## 7. Conclusions

Skeletal muscle progenitor cells arise from the PM and LPM that are heavily influenced by signals from the neural tube, notochord, ectoderm, and dorsal laminae. Combinatorial networks of signaling molecules regulate the expression of SSTFs that further regulate the commitment and differentiation to the myogenic lineage. The PM of the cranial domain forms early and gives rise to craniofacial, cardiac and vascular smooth muscles. PITX2^+^MSC^+^TBX1^+^ cells from the paraxial head develop the cardiogenic and head muscle cells. Head myogenesis is inhibited by WNT signaling. The segmented trunk PM forms the somites, in which the PAX3^+^ cells are influenced by WNT and SHH to commit to myogenic MYOD1^+^ cells and PAX7^+^ satellite stem cells.

## Figures and Tables

**Table 1:** Signaling molecules during myogenesis.

Gene	Anatomical Location	Model	Function	Reference
**Bone Morphogenetic Factor (BMP)**				
***Bmp2***		murine fibroblasts	prevention of myogenesis	[21,22]
		murine myoblasts	inhibition of *MyoG* expression	[23,24]
		fish	delayed muscle differentiation	[25]
***Bmp4***		human fetal myoblasts	regulation of proliferation	[26]
		chick	induction of embryonic and fetal myogenesis	[27]
		frog	induction of ventral mesoderm	[28]
		chick	inhibition of *Myod* and *Myf5*	[29]
		axolotl	somite development	[30]
***colloid-like 1*** BMP4 regulator	anterior somites	chick	neck muscle formation	[31]
***Noggin*** BMP4 antagonist	Neural Tube; Notochord	mouse	differentiation	[32,33]
		chick	growth inhibition	[34]
		mouse	fetal myogenesis; migration of muscle progenitor cells	[35,36]
***Bmp7***	dorsal Neural Tube	chick	muscle growth, apoptosis	[34]
**Fibroblast Growth Factor (FGF)**				
***Fgf2***	Central Nervous System	fish	inhibition of muscle pioneer cells	[19,24,25]
***Fgf4***	myotubes	mouse	mesoderm formation	[37]
		chick	inhibition of terminal differentiation of limb	[38]
		chick	induction of tendon-specific markers	[39]
		chick	patterning during gastrulation	[40]
		frog	activation of *Myod*	[41,42]
***Fgf5***	Lateral Plate Mesoderm	mouse	formation of germ layers	[43]
		chick	inhibition of myogenesis	[44]
***Fgf8***	caudal end of embryo	fish	terminal muscle differentiation	[45,46]
		chick	tendon formation	[39]
		fish	somitic muscle formation	[47]
		murine myoblasts	myotubes formation	[48]
		fly	mesodermal cell migration	[49]
		chick	mesodermal cell migration	[40]
		chick; mouse	myoblast proliferation	[50]
**Notch Pathway**				
***Notch***	Ectoderm	mouse	terminal muscle differentiation	[32,33]
		frog	inhibition of cell fusion; myotube formation	[51]
		mouse	cell fate decisions	[52]
		murine myoblasts	inhibition of muscle differentiation	[53]
***rbp-j*** NOTCH ligand	trunk somites	frog	determination of dorsolateral and ventral mesoderm	[28]
		axolotl	formation of the dermomyotome	[30]
		myeloma cells	inhibition of differentiation	[54]
		mouse	differentiation	[32,33]
		murine myoblasts	inhibition of differentiation	[55]
***Jagged-1*** NOTCH ligand	trunk mesenchyme, splachnopleure	mouse	differentiation	[32,33]
		murine myoblasts	inhibition of differentiation	[56,57]
***Delta1*** NOTCH ligand	Neural Crest cells	mouse	terminal differentiation	[32,33]
		chick	inhibition of terminal differentiation	[58]
		chick	terminal differentiation, inhibition of exiting the cell cycle	[59]
***Mesp2*** NOTCH ligand	Presomitic Mesoderm	mouse	somitic boundaries	[60]
		mouse	cellular epithelialization	[61]
		mouse	somitogenesis; rostro-caudal polarity	[62]
		mouse	inhibition of NOTCH targets	[63]
**Ephrin Ligands**				
***EphA4***	Presomitic Mesoderm	mouse	somitogenesis	[61]
		chick	somitogenesis	[64]
***ephinb2***	somites	chick	somitogenesis, expression of N-cadherin	[64]
***Ripply***	Presomitic Mesoderm	mouse	rostro-caudal polarity	[62]
		mouse	*Mesp2* expression	[65]
***neurotrophin 3***	Neural Tube	chick	*Pax3* expression induction, somitic myogenesis regulation	[66]
		chick	dermatome dissociation; epithelial-mesenchymal transition	[67]
**Retinoic Acid (RA)**				
***Ra***	cranial end of the embryo	mouse	*Myf5* expression	[68]
		fish	somitogenesis; fast muscle differentiation	[69]
		murine myoblasts	inhibition of myoblast proliferation; differentiation	[69]
		mouse	somitogenesis	[70]
**Sonic Hedgehog (SHH)**				
***Shh***	Neural Tube, Notochord	chick	somitogenesis	[66]
		fish	formation of slow muscle precursor cells	[71]
		chick	induction of myogenesis	[72]
		fish	inhibition of *pax3/7*; activation of *myf5* and *myod*	[47]
		chick	induction of *pax3* and *myod1*	[73]
		chick	activation of slow MyHC	[74]
		mouse	epaxial and hypaxial myogenesis	[75]
**Wnt**				
***Wnt1***	Neural Tube	chick	*pax3* activation; somitogenesis	[66]
		murine myoblasts	formation of slow fiber types promotion	[76]
		chick	formation of paraxial mesoderm	[72]
		mouse	Formation of medial and dorsal portion of somites	[77]
		mouse	*Myf5* activation	[15]
***Wnt3a***	Neural Tube, Apical Ectodermal Ridge (AER)	murine myoblasts	*Bmp4* activation; formation of slow fibers	[76]
		mouse	Formation of dorsomedial part of somites	[77]
		murine myoblasts	myotube formation	[78]
		human embryonic stem cells	myogenic commitment	[79]
		murine myoblasts	myotube formation	[80]
		pluripotent stem cells	cardiomyocytes proliferation	[81]
***Wnt4***	Neural Tube	chickmurine myoblasts	activation of *pax7* and *myod1*; formation of fast myofibers	[82]
		mouse embryos	activation of *Myf5* and *Myod1*	[15]
		murine myoblasts	myotube formation	[78]
		murine satellite cells and myoblasts	activation of myogenesis	[83]
***Wnt6***	Paraxial Ectoderm	mouse	activation of *Myf5* and *Myod1* in paraxial mesoderm	[15]
		chick	activation of *Pax3*, *Paraxis*, *Myf5*, *Myog*, *Desmin* and MyHC	[84]
***Wnt7a***	Dorsal Ectoderm	mouse	activation of *Myod1*	[15]
***Wnt11***	somites	chick	formation of fast myofibers	[85]
		chick	elongation of myocytes	[86]
